# Supplementary material for: Parallel reverse genetic screening in mutant human cells using transcriptomics
Source: Mol Syst Biol. 2016 Aug 1;12(8):879. doi: 10.15252/msb.20166890 (PMC5119491; doi:10.15252/msb.20166890)
Supplement: Supplementary file 1 — Appendix [file MSB-12-879-s001.pdf]

Appendix:

Parallel reverse genetic screening in mutant human cells using transcriptomics

Bianca V Gapp, Tomasz Konopka, Thomas Penz, Vineet Dalal, Tilmann Burckstummer, Christoph Bock and Sebastian MB Nijman

Contents

Figure S1. Collection of CRISPR/Cas9 knock-out cell lines **2**

Figure S2. Hypoxic-like state as a passenger effect in screened clones **3**

Figure S3. Technical covariates affecting measurement of stimulus response **4**

Figure S4. Summary of scripts used for analyses and visualizations **5**

The Human Kinome & HAP1 knock-out cell lines

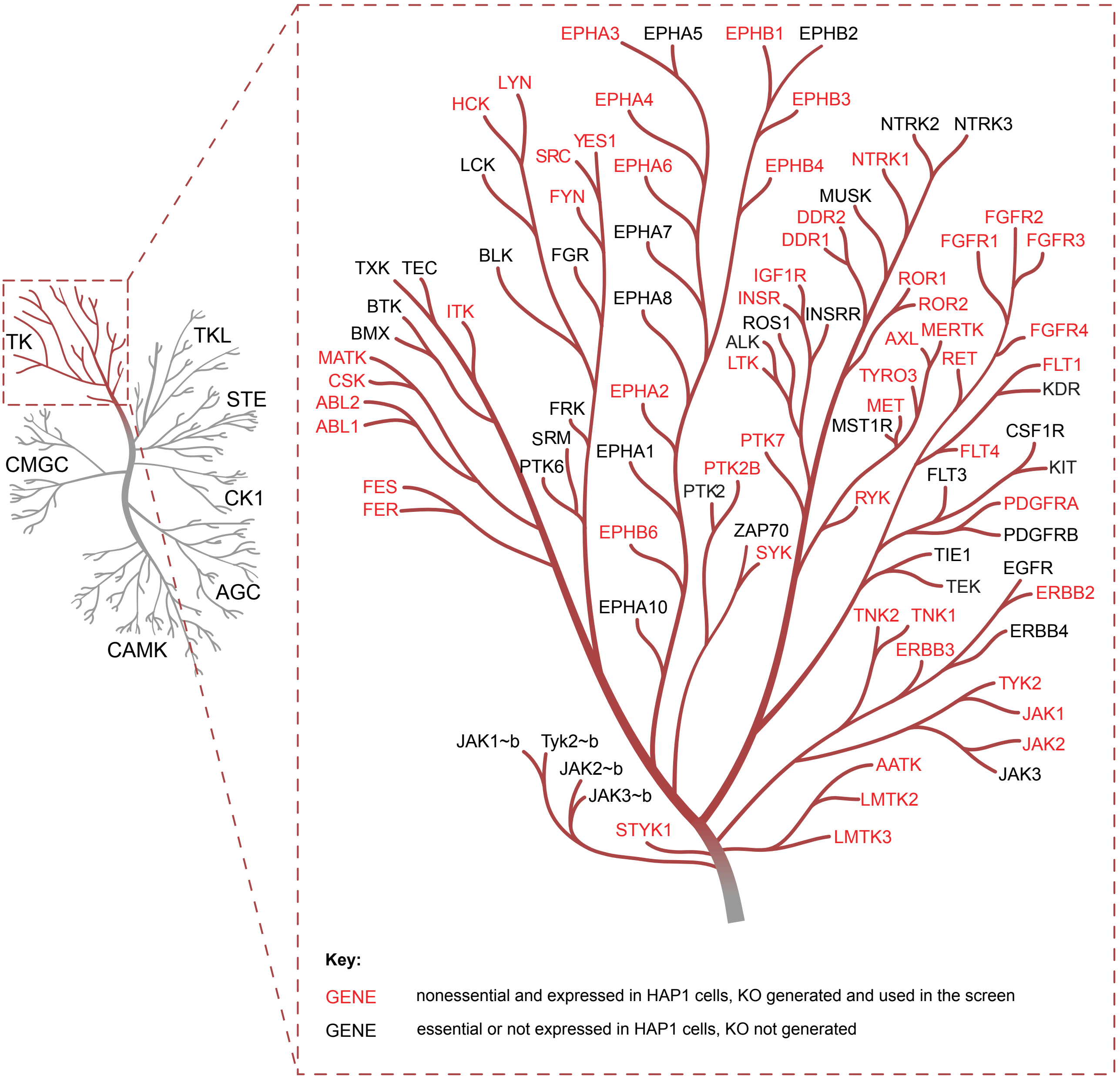

Figure S1: **Collection of CRISPR/Cas9 knock-out cell lines covering the tyrosine branch of the human kinome.** On the left is the human kinome phylogenetic tree; abbreviations near the branches indicate kinase sub-families. The detailed view on the right shows the tyrosine kinase branch. Red labels indicate tyrosine kinases that are expressed and non-essential (see Methods section) in HAP1 cells. Black labels indicate tyrosine kinases not selected for CRISPR/Cas9 editing.

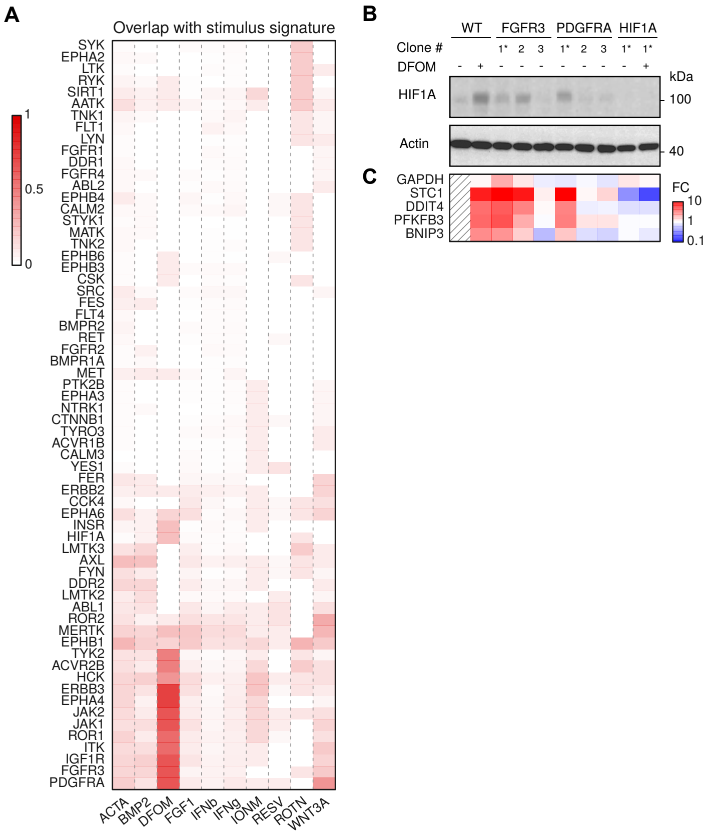

Figure S2: **Hypoxic-like state as a passenger effect in screened clones.** (A) Comparison of transcriptional profiles of unstimulated knock-out cells with stimulation gene sets. Knock-out cell lines are clustered based on overlap between profiles. (B) Protein expression of HIFA in WT and KO cells. Asterisks label clones used in the reverse genetic screens, i.e. FGFR3-KO and PDGFRA-KO clones displayed in previous panel. Actin was used as a loading control. WT, wild type; KO, knock-out. (C) qRT-PCR validation of DFOM signature gene expression in WT and KO cells. qRT-PCR measurements for five genes in wildtype cells were used as reference points. Measurements in other cell lines were then used to compute fold-change with respect to the reference levels. GAPDH is a housekeeping gene and shows little change across conditions. The other genes are DFOM signature genes upregulated upon DFOM stimulation and in selected KO clones.

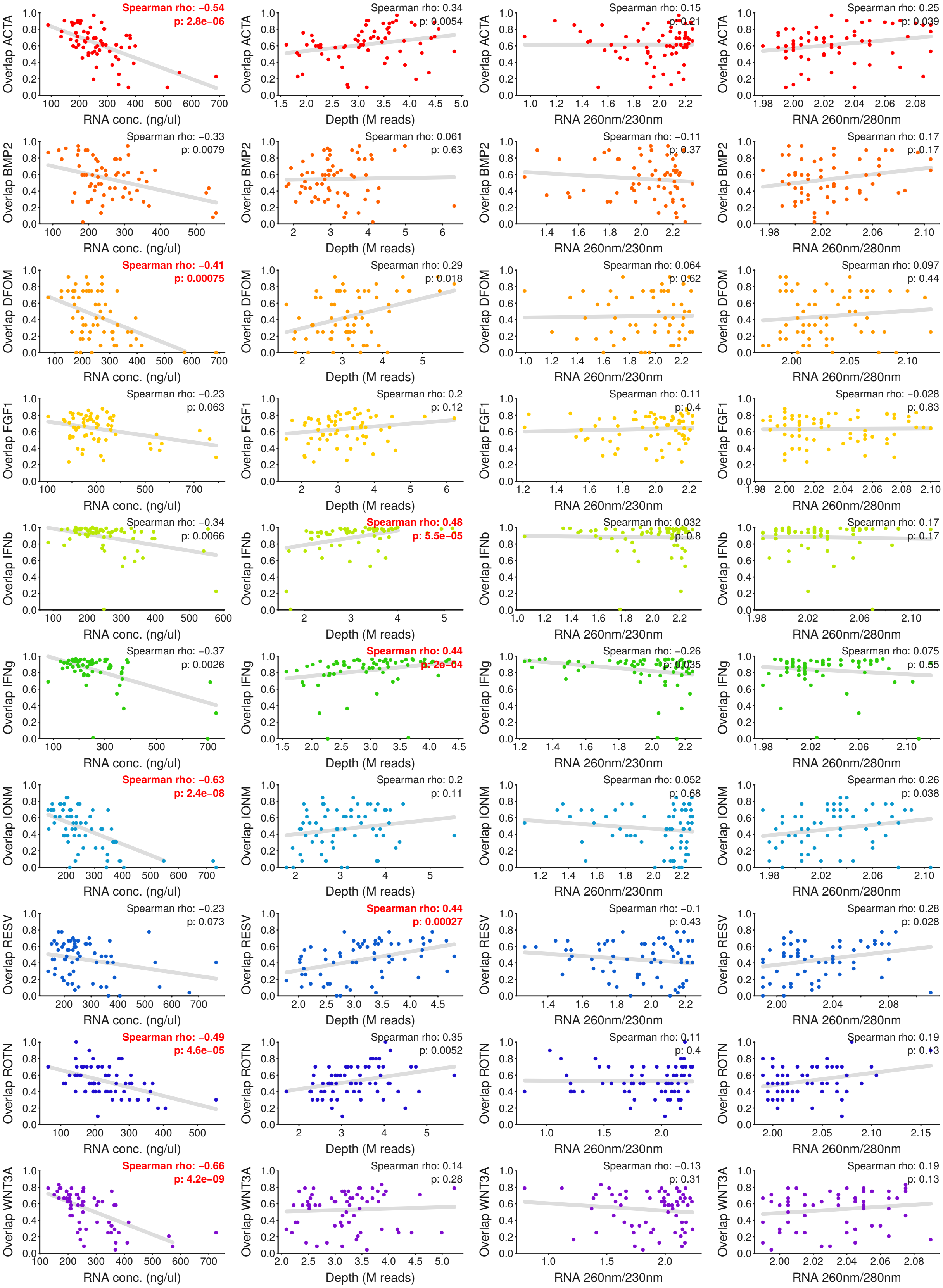

Figure S3: **Technical covariates affecting measurement of stimulus response.** Dependency of gene signature overlap (wild type and knock-out signatures for each stimulus) on RNA concentration, sequencing depth, and RNA quality (light absorbance at 260nm vs 230nm, and 260nm vs 280nm). Titles marked in red suggest significant correlations.

Computational Analysis

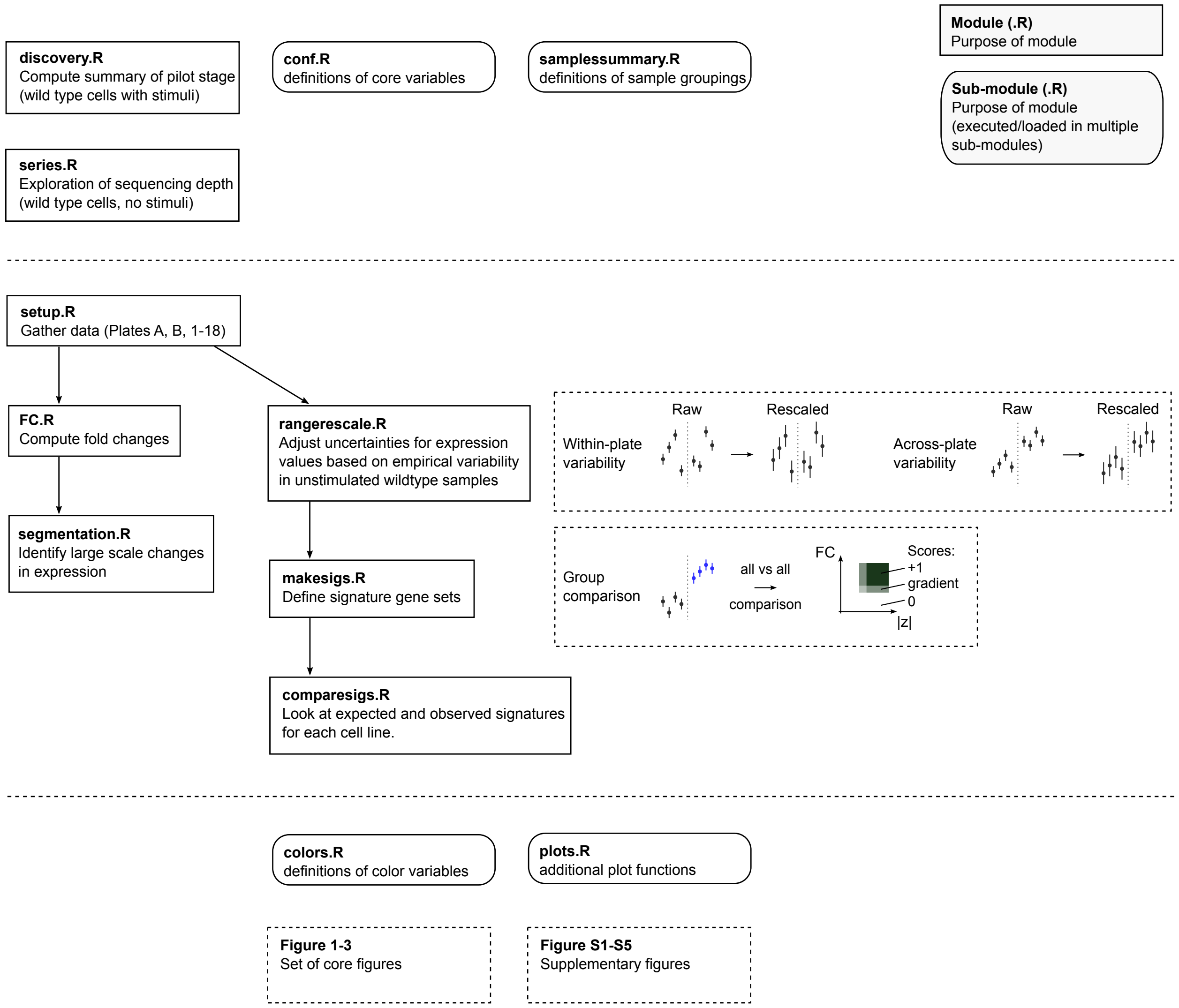

Figure S4: **Schema of analysis modules used to produce analysis results and visualizations.** Each box represents a distinct module/script and the flowchart shows dependencies and order of execution. All necessary input data and module code are available online at doi:10.5281/zenodo.51842. A README file describes R package dependencies, all of which can be installed through CRAN or github.
