# Supplementary material for: Parallel reverse genetic screening in mutant human cells using transcriptomics
Source: Mol Syst Biol. 2016 Aug 1;12(8):879. doi: 10.15252/msb.20166890 (PMC5119491; doi:10.15252/msb.20166890)
Supplement: Supplementary file 2 — Expanded View Figures PDF [file MSB-12-879-s002.pdf]

Expanded View Figures

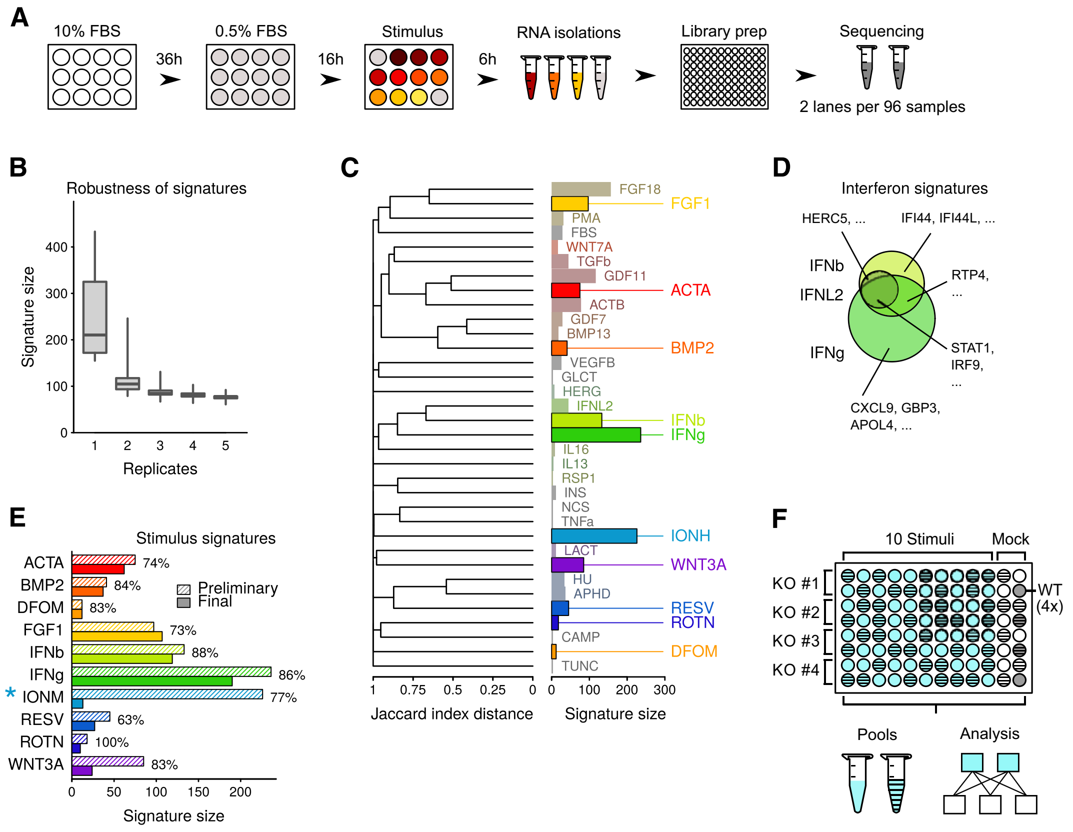

Figure EV1. Gene signatures of wild-type cells upon stimulation.

A Experimental design of stimulations in HAP1 cells. HAP1 cells were seeded in 12-well plates, cultured for 36 h in standard medium (10% FBS) and 16 h under reduced serum conditions (0.5% FBS). Following stimulation for 6 h, total RNA was isolated and libraries prepared for next-generation sequencing. A total of 48 samples were pooled for sequencing on each lane of an Illumina HiSeq 2000 sequencer. FBS, fetal bovine serum.

B Modeling of robustness of gene signatures with increasing replicate number based on a set of eight replicates of FGF18 stimulation experiments. For each number of replicates on the *x*-axis, we selected subsets of the eight replicates and computed the signature size with respect to a set of wild-type controls. This calculation was repeated 20 times. Boxplots show quartiles (minimum, 25, 50, 75%, and maximal) results among the repeated bootstrap operations.

C Visualization of stimulus signatures in HAP1 cells. Bar charts are as in Fig 1C. Similarity between signatures is determined by the Jaccard index distance (1—Jaccard index between signature sets), and the dendrogram is produced using a hierarchical complete agglomeration method.

D Comparison of gene signatures in IFNb, IFNL2, and IFNg stimulated HAP1 cells. Examples of signature genes specific and shared between the three stimuli are indicated.

E Signature size for the ten selected stimuli. Preliminary (2 replicates) and final (3 or more replicates) signature sizes are indicated. Percentages indicate overlap between preliminary and final signatures (size of intersection divided by size of final set). Asterisk next to IONM indicates that the different signature size is affected by a change in stimulus concentration between preliminary and final experiments.

F Design and data analysis layout for the reverse genetic screening phase. Library preparations were performed in a 96-well format. Each library preparation batch consisted of four knockout cell lines with ten stimuli, mock treatment, and four unstimulated wild-type controls. A total of 48 samples were pooled and sequenced on one lane of an Illumina 2000 sequencer. Genes were scored as differentially expressed by comparing replicates of stimulated and unstimulated samples from the same library preparation batch. WT, wild type; KO, knockout.

 © 2016 The Authors

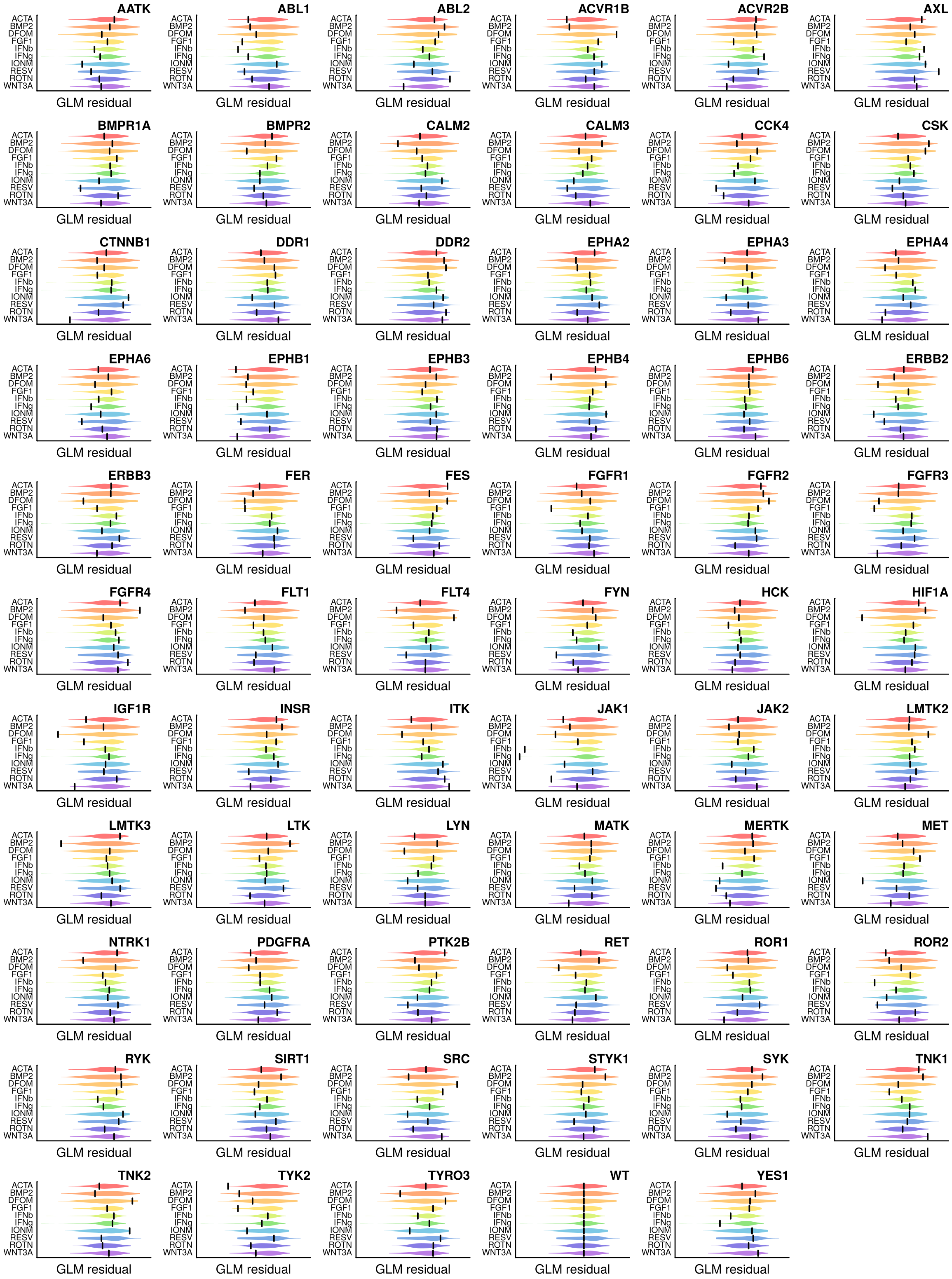

Figure EV2. Overview of mutant cell lines' response to stimulation.
Matrix of response visualizations for all screened cell lines. In each panel, violins depict distributions of response scores from the entire mutant panel. Scores are overlaps of signature gene sets with expected signature sets, corrected for technical variables using general linear models (GLMs). Bars display response of the mutant cell line indicated in the panel title.

© 2016 The Authors

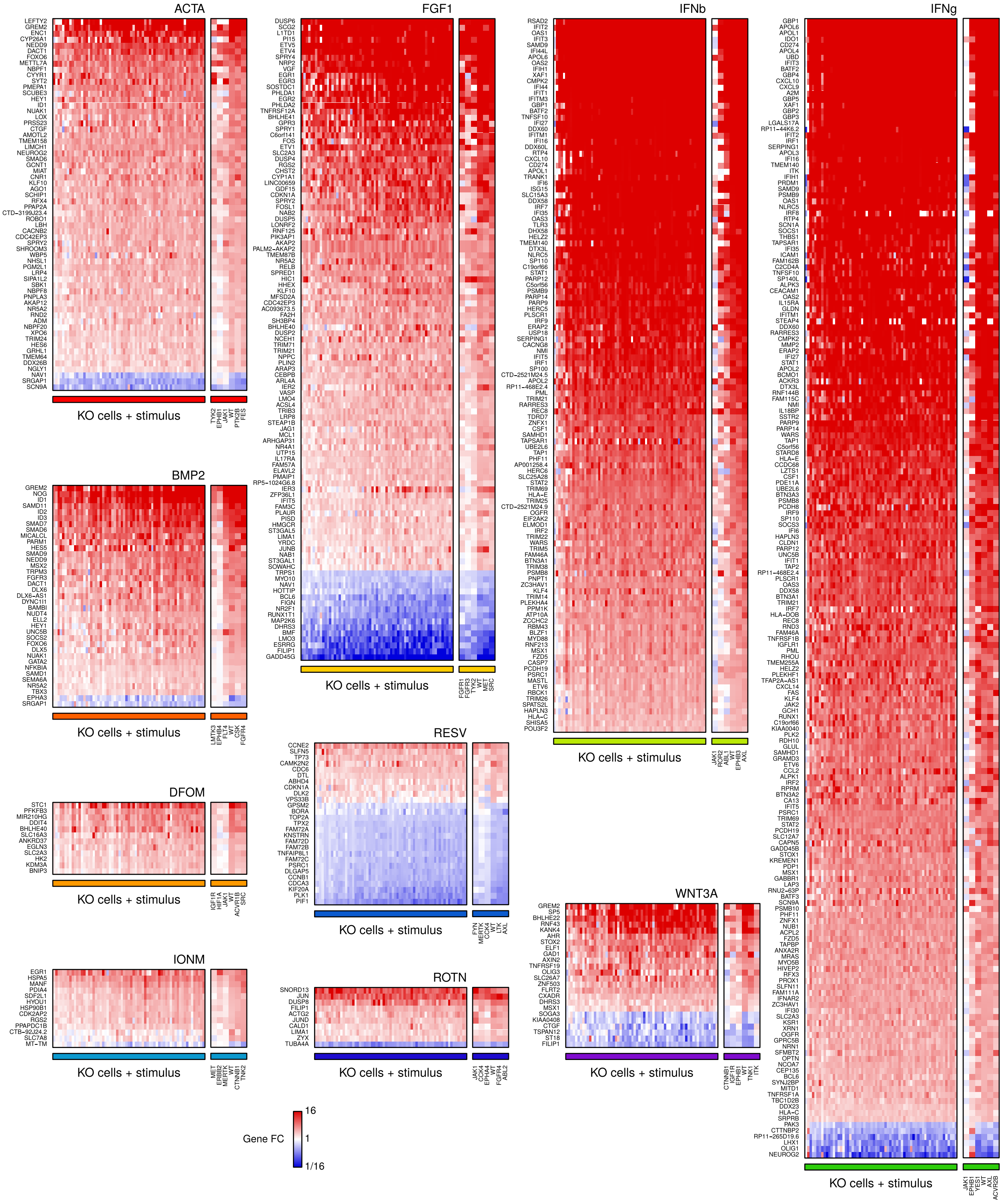

Figure EV3. Expression changes in signature genes.

Heatmaps depicting fold changes in KO cell lines upon stimulation relative to their unstimulated level. Genes part of the stimulus signatures are labeled on the vertical axes. The horizontal axes consist of WT and KO cell lines; cells are ordered differently in each heatmap according to their stimulus response score. Small heatmaps on the side provide a detailed view of three KO lines with the smallest stimulus response scores, WT cells, and two KO lines with the largest stimulus response scores. FC, fold change; WT, wild-type; KO, knockout.

 © 2016 The Authors

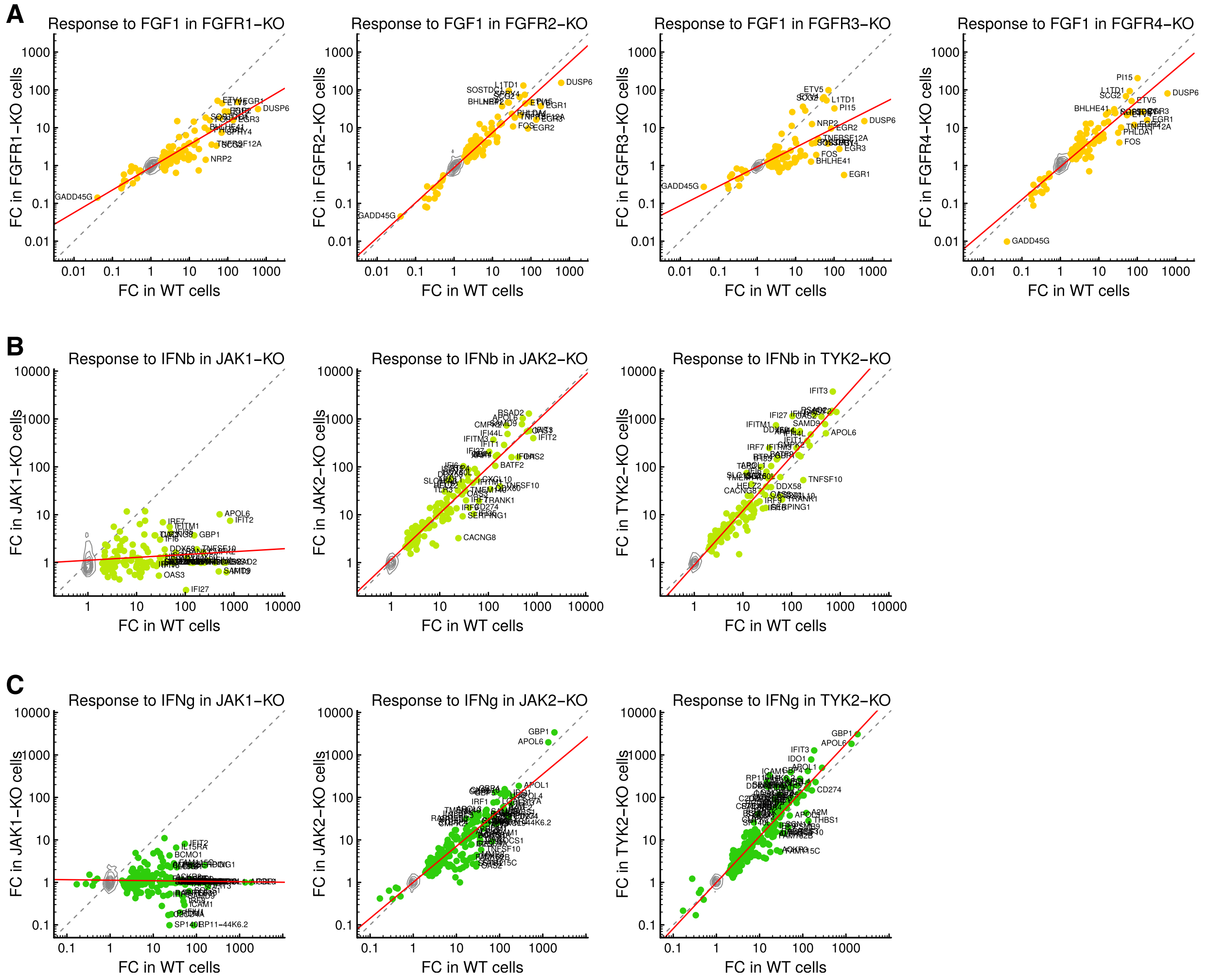

Figure EV4. Gene signatures of selected mutants.

A Comparison of response signatures in wild-type and FGFR family mutant cells. Contours depict genes not differentially expressed; dots indicate FGF1 signature genes; gray dotted line is the diagonal of equal response; red line is a linear fit using signature genes. FC, fold change; WT, wild-type; KO, knockout.

B Same as in (A) except for JAK family mutants and IFNb stimulation.

C Same as in (B) except for IFNg stimulation.

© 2016 The Authors

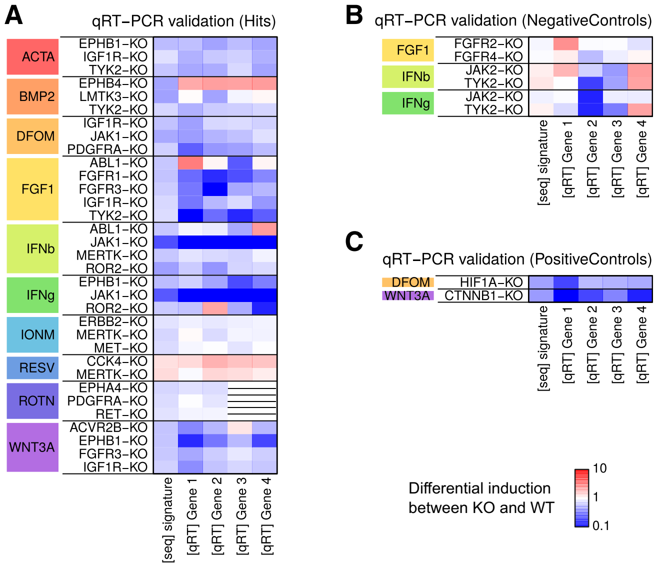

Figure EV5. qRT–PCR validation of selected mutants.

A Heatmap of differential induction of signature genes upon stimulation. Each row represents an independent experiment in which WT and KO cells were stimulated, and the expression of four genes measured using qRT–PCR. Similar induction levels in the four genes and in the RNA-seq signature conveys concordant results between the PCR and sequencing assays. WT, wild-type; KO, knockout.

B Same as in (A) except for KO and stimulus combinations for expected negative interactions.

C Same as in (A) except for KO and stimulus combinations for expected positive interactions.

© 2016 The Authors
