# Supplementary material for: Parallel reverse genetic screening in mutant human cells using transcriptomics
Source: Mol Syst Biol. 2016 Aug 1;12(8):879. doi: 10.15252/msb.20166890 (PMC5119491; doi:10.15252/msb.20166890)
Supplement: Supplementary file 3 — Table EV1 [file MSB-12-879-s003.zip › TableEV1/README.rtf]

TableEV1:Summary of the polyptides and small molecules used for HAP1 stimulations.
